# Intranasal dexmedetomidine sedation for EEG in children with autism spectrum disorder

**DOI:** 10.3389/fpsyt.2024.1462526

**Published:** 2024-10-10

**Authors:** Arianna De Laurentiis, Chiara Pastori, Carmela Pinto, Stefano D’Arrigo, Margherita Estienne, Sara Bulgheroni, Giulia Battaglia, Marco Gemma

**Affiliations:** ^1^ Department of Pediatric Neurosciences, Fondazione Istituto di Ricovero e Cura a Carattere Scientifico (IRCCS) Istituto Neurologico Carlo Besta, Milan, Italy; ^2^ Epilepsy Unit, Fondazione Istituto di Ricovero e Cura a Carattere Scientifico (IRCCS) Istituto Neurologico Carlo Besta, Milan, Italy; ^3^ Neuroanesthesia and Intensive Care Unit, Department of Neurosurgery, Fondazione IRCCS Istituto Neurologico Carlo Besta, Milan, Italy

**Keywords:** autism, EEG, sedation, dexmedetomidine, behavioral disturbance

## Abstract

**Introduction:**

The aim of the study was to assess the efficacy of In-Dex sedation in comparison to oral melatonin and hydroxyzine in individuals with Autism Spectrum Disorder (ASD) undergoing EEG recording and 15 determine which categories of patients exhibit the most favorable response to In-Dex sedation.

**Methods:**

This retrospective observational study involved pediatric patients with ASD who underwent sleep-EEG recording across two periods, before (biennium 2018-19) and after (biennium 2021-22) the routine implementation of In-Dex sedation. Clinical, EEG, and sedation data were stored in a database. A logistic multiple regression model was employed, with the failure of EEG serving as the dependent variable.

**Results:**

In the first period 203 EEGs were performed with a rate of failure of 10.8%, while in the second one 177 EEGs were recorded with a percentage of failure of 7.3% (8.3% with MH 23 sedation and 5.8% with In-Dex sedation). No significant adverse events were reported in either period. Multivariate logistic analysis demonstrated that In-Dex decreased the probability of failure (OR=0.25, 25 (0.61-0.88)), while the presence of behavioral disturbances (OR=3.65((1.54-8.85)) and the use of antipsychotic drugs (OR=2.76, (1.09-6.95)) increased it.

**Discussion:**

In the light of these results, we can state that In-Dex sedation is safe and reduce EEG failure rate compared to the use of melatonin and hydroxyzine alone, particularly in patients with severe behavioral issues.

## Introduction

ASD (Autism Spectrum Disorder) is a neurodevelopmental disorder characterized by persistent challenges with social behavior and communication, restricted interests, and repetitive behavior (1).

It is well documented that patients with ASD have an increased risk of having epilepsy compared with general population. Data on epilepsy prevalence in this condition reported in the literature are heterogenous. Meta-analysis incorporating studies from 1963 to 2006 reported a prevalence of 21.5% in autistic subjects with intellectual disability (ID) versus 8% in patients without ID (2). Another recent systematic review on the topic, including data from 74 studies involving more than 283,000 people, found a median overall prevalence of epilepsy in ASD of 12.1% (3).

The rate of occurrence of interictal epileptiform discharges (IEDs) in ASD has been reported in 6 and 30% of cases, up to 65% in some studies (4–7). Monitoring the IEDs presence and development in children without clear clinical seizures, could help to improve phenotyping of ASD individuals, to manage single patients and to understand the possible implication of IEDs in ASD pathogenesis (4, 5, 8).

Therefore, electroencephalogram (EEG) is highly recommended in the workup of this condition. However, obtaining studies of adequate quality is often challenging due to the presence of aberrant behaviors and poor adaptive skills related to the condition. In children with profound agitation, severe irritability, aggression, tactile aversion, and difficulties with transition from one environment to another, a pharmacological sedation may be necessary to successfully perform an EEG.

On the other hands, some sedative drugs commonly used for pediatric sedation significantly affect the EEG pattern. Propofol, an agonist of GABAA receptor, induces sedation by enhancing GABA-mediated inhibition of pyramidal neuron. The EEG is characterized by an abrupt anteriorization of alpha rhythms and an increase of incoherent slow oscillation. Previous studies have indicated that propofol can cause various changes in the EEG tracing, including the suppression of critical activity and the induction of epileptiform abnormalities (9).Ketamine, an NMDA antagonist, induces unconsciousness by blocking excitatory glutamatergic neurons. Ketamine-induced anesthesia has been shown to be associated with a high-frequency EEG pattern that resembles wakefulness more than sleep. Because of its favorable safety profile, it has been included in some protocols as a non-first-choice sedative agent for EEG (10). Oral chloral hydrate has widely been used in the past because it exhibits minimal interference with the EEG. However, it has disadvantages such as long duration of action, prolonged recovery time, potential significant adverse effects, and limited efficacy, especially in case of uncooperative patients (11). Similarly, midazolam has been used as a pre medication agent for EEG recording in children. However, potential adverse reactions such as paradoxical reactions, respiratory depression and the risk of over‐sedation, make it not and ideal medication (12).

In our hospital, sedation and sleep induction for EEG recording in children with ASD have been traditionally accomplished through sleep deprivation and the use of oral melatonin and hydroxyzine. Hydroxyzine is a long acting first generation H1 antagonist with central nervous system depressant activity which has been used safely and effectively for sleep induction in pediatric EEG recordings (11). This approach has some important limitations since it requires patient cooperation to warrant a correct drug intake by the oral route. Moreover, traditional sedatives may not be effective in subjects with ASD, even with appropriate intake of the prescribed dose.

In recent years, many studies have highlighted the usefulness of Dexmedetomidine for pediatric sedation in EEG studies. Compared to traditional sedation methods, it offers several important advantages in this setting. Dexmedetomidine is an agonist of central adrenergic α-2 receptors which can be effectively delivered by various routes: intravenous, oral, intranasal, and intramuscular. The intranasal delivery is particularly appealing, since it is painless, well tolerated and provides a good systemic absorption via the wide nasal capillary bed. Moreover, compared to the oral route, it has the advantage of being more effectively administered to patients with low levels of cooperation, such as children with ASD. Furthermore, the intranasal route is not only effective but is often more acceptable to both parents and patients compared to the intravenous route.

Previous studies have shown that Dexmedetomidine has minimal effects on the basic background waves of the brain and does not hinder interpretation of the EEG. In children it produces an EEG pattern similar to Stage II sleep, with modest increases in theta, alpha, and beta activity, but without affecting the detection of epileptic discharges (13, 14).

To date, few studies have explored the effectiveness of intranasal Dexmedetomidine (In-Dex) as a sedative agent for EEG testing in pediatric patients with ASD (15, 16). Given the unique challenges in managing these patients, particularly due to severe behavioral difficulties, it is essential to conduct targeted research focusing on this specific subpopulation. While In-Dex shows promise based on its characteristics, additional evidence is required to confirm its efficacy in children with ASD.

The principal endpoint of our study is to compare overall sedation effectiveness in children with ADS undergoing EEG recording before and after the introduction of intranasal Dexmedetomidine in our clinical practice. As secondary endpoint, we investigated subgroups of patients presenting with different clinical features, to identify the best responders to In-Dex sedation in this setting.

## 2 Methods

### 2.1 Participants

We retrospectively analyzed clinical, EEG and sedation data of ASD inpatients submitted to sleep-EEG recording at the Department of Pediatric Neurosciences of Fondazione IRCCS Istituto Neurologico Carlo Besta; two periods were considered, before (January 2018 - December 2019) and after (January 2021 - December 2022) the routine introduction of In-Dex sedation during EEG recording. Data from 2020 were excluded from our analysis, since few ASD patients underwent EEG in that period due to the COVID-19 pandemic. Patients younger than 18 years diagnosed as ASD and submitted to sleep-EEG recording in our Institute were included in the study.

The study complied with the general ethical requirements for retrospective observational studies. In particular, no experimental interventions were performed and patient identity cannot be retrieved from the manuscript. In-Dex sedation is considered part of ordinary clinical practice and a written informed consent for sedation was obtained from the parents of each participant. For this type of study, an ethical approval was not required.

### 2.2 Evaluation protocol

The following variables were recorded for each patient in a dedicated database: gender, age, intellectual level, presence of significant behavioral disturbances, current use of antipsychotic drugs. The intellectual level was evaluated based on clinical examination, individualized standardized cognitive testing, and assessment of adaptive functioning, according to DSM-5 criteria, and categorized as a binomial variable into: 0 = normal/borderline cognitive functioning or mild Developmental Delay/Intellectual Disability (DD/ID), 1 = moderate to severe DD/ID. Significant behavioral disturbances defined by the presence of severe irritability, agitation, self-injurious behaviors, temper outbursts/tantrums were categorized as: 0 = absence of significant behavioral problems, 1 = presence of significant behavioral problems. The relevant information was derived from medical history and detailed patient description as reported in clinical records.

### 2.3 Sedation

During the 2018-2019 study period all patients were sedated with an oral solution of melatonin (1-3 mg) and hydroxyzine (0.5-1 mg/Kg) (MH sedation) 30 minutes before starting the EEG recording. Parents were encouraged to partially sleep-deprive their child over the night before the EEG registration. Fasting was not recommended before MH sedation. Anesthesiologists were not involved in MH sedation.

In-Dex administration through both nostrils (total dose 5 mcg/Kg, up to a maximum of 200 mcg) was introduced in January 2021. Pre-sedation fasting was set at 6 hours for solid food and 2 hours for clear fluids. Throughout the procedure, continuous SpO2 and ECG monitoring was maintained in the presence of an anesthesiologist experienced in pediatric sedation. The pediatric neurologist in charge for the patient was responsible for the decision on the use of either MH or In-Dex sedation; the latter was proposed for cases with a lower level of collaboration. The EEG (10-20 montage) completed by the polygraphic recording of the electrooculogram, the electrocardiogram and the pneumogram was scheduled 30 minutes after treatment (during sedation) and was monitored for 45 minutes.

After completion of the EEG procedure, all patients were kept under observation in a dedicated room by a trained nurse; ECG and SpO2 monitoring was maintained until a modified Aldrete score (17) of 10 was achieved and the neurological clinical status returned to baseline. Possible adverse events during the sedation procedure were recorded if they required any intervention by the attending anesthesiologist.

### 2.4 Data analysis and statistics

Continuous variables are reported as mean ± SD[median(IQR)]. Categorical variables are reported as number (percentage).

Statistical analysis was performed with R Core Team (2023). _R: A Language and Environment for Statistical Computing_. R Foundation for Statistical Computing, Vienna, Austria. https://www.R-project.org/.

Due to the retrospective, non-randomized study design of our series, our main purpose was descriptive. Nevertheless, a logistic multiple regression model was suggested to evaluate a possible effect of In-Dex on the failure of the EEG recording.

“Failure of the EEG recording” was kept as the outcome dichotomous variable of interest and of “In-Dex” as the independent dichotomous variable of interest. Five other variables were also evaluated as candidate covariates affecting the model in a univariate variable selection process, namely age, gender, intellectual level, presence of significant behavioral disturbances, and current use of antipsychotic drugs, as described above (see Study population). This univariate analysis was accomplished with a generalized linear model (base-R function “glm”) specifying a “binomial” error distribution and a “logit” link function, as appropriate for logistic regression. As standard practice, to help avoid artifactual significance, variables yielding a Likelihood Ratio test p<0.25 were considered as candidates for a possible final model. Variables in the resulting multivariate model were removed from the model one by one starting with the one with the highest p-value. In building the final model, p< 0.05 was considered statistically significant and the model with the lowest AIC (Akaike Information Criterion) was preferred.

The linearity assumption of continuous predictors was checked by visually inspecting the scatter plot between each predictor and the logit values.

In order to verify possible non-additive effects over and above the effect of the linear effects of the single predictors, the effect of interactions between variables entering the final model were evaluated by the standard method of adding product variables to the model with a procedure consistent with the variable selection method described above.

Each variable included in the final model were checked for multicollinearity, i.e. a possible high correlation with other independent variables that influences its marginal contribution. This was accomplished by evaluating the variance inflation factor (VIF) (R function “car:vif”), which measures how much the variance of each coefficient is inflated due to multicollinearity in the overall model. VIF values ≥5 would suggest multicollinearity.

We calculated the whole-model p-value by comparing the final model (considered the “full model”) with a reduced “null model” (basic-R function “anova”).

The match between observed and expected outcome was tested as a goodness-of-fit measure with the Hosmer-Lemeshow goodness-of-fit p-value (R function “glmtoolbox:hltest”).

Odds ratios (ORs) are reported as OR (95% CI) where the CIs were computed with the Wald method.

Although some of the EEG recordings comprising the study data may have reflected more than one procedure from an individual patient, quantities observed during different procedures were assumed to constitute statistically independent observations for the purpose of data analysis.

## 3 Results

**Tables 1**, **2** summarize demographics and clinical characteristics of patients enrolled in the first (2018-2019) and in the second (2021-2022) biennium, respectively. **Table 3** shows numbers of EEG recordings, rate of failures and presence of epileptiform discharges in both periods, considering separately MH and In-Dex sedations.

**Table 1:** Demographics and clinical characteristics of patients enrolled in biennium 2018-2019.

Total patients	203
Gender	
- Male - Female	170 (83.7%) 33 (16.3%)
**Age**	13 months – 156 months (58 ± 28)
Intellectual functioning	
- Normal or mild DD/ID - Moderate to severe DD/ID	72 (35.5%) 131 (64.5%)
Behavioral disturbance	
− No − Yes − + pharmacological treatment	132 (65.0%) 71 (35.0%) 24 (11.8%)
Seizures	
- Yes - No	193 (95.1%) 10 (4.9%)

**Table 2:** Demographics and clinical characteristics of patients enrolled in biennium 2021-2022.

Total patients	172
Gender	
- Male - Female	148 (86.0%) 24 (14.0%)
**Age**	22 months – 192 months (70 ± 40)
Intellectual functioning	
- Normal or mild DD/ID - Moderate to severe DD/ID	49/172 (28.5%) 123/172 (71.5%)
Behavioral disturbance	
− No − Yes − + pharmacological treatment	89 (51.7%) 83 (48.3%) 16 (9.3%)
Seizures	
- Yes - No	159 (92.4%) 13 (7.6%)

**Table 3:** EEG recordings, rate of failures and presence of epileptiform discharges in each biennium.

	Biennium 2018-2019			Biennium 2021-2022		
	Total	MH sedation	In-Dex sedation	Total	MH sedation	In-Dex sedation
**EEGs performed**	203	203/203 (100.0%)	0	177	108/177 (61.0%)	69/177 (39.0%)
**EEGs failed**	22/203 (10.8%)	22/203 (10.8%)	0	13/177 (7.3%)	9/108 (8.3%)	4/69 (5.8%)
**Epileptiform abnormalities**	43/181 (23.8%)	43/181 (23.8%)	0	78/164 (47.6%)	50/99 (50.5%)	28/65 (43.1%)

### 3.1 Biennium 2018-2019

In this first 2-year period, 203 patients were enrolled: 170 (83.7%) were males. Age ranged from 13 months to 176 months; 58 ± 28 [53 (21)]. With respect to intellectual functioning, 72 (35.5%) children had normal/borderline level or mild DD/ID, while 131 (64.5%) presented a moderate to severe DD/ID. Behavioral disturbances were present in 71 (35.0%) patients, and 24 (11.8%) were treated with antipsychotic drugs.

All patients received MH sedation and no adverse event requiring anesthesiologic intervention occurred. The EEG examination could not be successfully completed in 22 (10.8%) cases due to inadequate sedation; patients either didn’t fall asleep or only achieved light and unstable sleep with significant movements, which impaired EEG recording and subsequent evaluation. The EEG recording demonstrated epileptiform discharges in 43 (23.8%) of the 181 children who successfully completed the exam.

### 3.2 Biennium 2021-2022

In the second period, 172 patients were enrolled: 148 (86.0%) were males; their age ranged from 22 months to 192 months; 70 ± 40 [57 (43)]. Intellectual functioning was normal/borderline or mild DD/ID in 49 (28.5%) children, whereas 123 (71.5%) presented a moderate to severe DD/ID. Behavioral disturbances were reported in 83 (48.3%) patients, and 16 (9.3%) were treated with antipsychotic drugs. Five patients were submitted twice to the EEG examination; overall, 177 EEGs were performed: 108 (61%) with MH sedation, and 69 (39%) with In-Dex sedation. The 5 patients, who had to repeat the EEG examination, received first unsuccessful MH sedation and were rescheduled for In-Dex sedation, which were successful. Overall, the EEG recording could not be completed in 13 (7.3%) cases: 9/108 (8.3%) with MH sedation and 4/69 (5.8%) with In-Dex sedation. In all these EEG recordings, failure was caused by inadequate sedation characterized by wakefulness or light sleep with movement artifacts that impaired EEG evaluation. No adverse event requiring anesthesiological intervention occurred.

The EEG recording demonstrated epileptiform discharges in 78 (47.6%) of the 164 successfully completed procedures: 50/99 (50.5%) in the MH sedation subgroup and 28/65 (43.1%) in the In-Dex sedation subgroup.

### 3.3 EEG failure analysis

Three independent variables entered a multivariate logistic model for failure of the EEG procedure: In-Dex sedation reduced the chance of failure [OR=0.25(0.61-0.88)], whereas the presence of significant behavioral disturbances [OR=3.65(1.54-8.85)] and current use of antipsychotic drugs [OR=2.76(1.09-6.95)] increased it. Although non-significant, the biennium of the procedure was kept in the model as a correction factor (OR=1.04(0.42-2.42).

No interaction coefficient significantly affected the outcome variable, so that no action was necessary to model interaction as predictors. All variables in the final model exhibited a VIF < 1.5, hence collinearity could be ruled out. The whole model P value was <0.001 and the Hosmer-Lemeshow goodness-of-fit test P was 0.80. **Supplementary Table S4** in the **Supplementary Material** section reports on the details of variable selection and the final model.

Given the significant effect in the final model of the current use of antipsychotic drugs, a possible effect of the duration of such therapies was evaluated in the 41 patients receiving antipsychotics. Although a trend was apparent favoring EEG failure in patients with longer therapy (7.23 ± 8.84[4(0.5-12)] versus 3.24 ± 8.24[0.5(0.5-2.20)] months), this difference was not significant (P=0.223)and was not considered in the predictive model.

## 4 Discussion

Several Authors highlighted the complexity of managing diagnostic procedures in children with ASD. Moreover, given the raising prevalence of this condition in recent years (18), the need to obtain reliable EEG evaluations is increasingly common for clinicians. ASD exhibits a variety of clinical presentations, which makes the statement of well-defined guidelines a challenging task. Difficulties in verbal and nonverbal communication, resistance to changes in routine and environmental transition, sensory abnormalities, and other specific clinical features, are common in ASD and may present in daily life as irritability, aggression, and other dysfunctional behaviors. These features are differently expressed by ASD patients and variably impact the degree of cooperation during diagnostic procedures or physical examinations.

Sleep-EEG recording is an important element of the neurological assessment of children with ASD. This retrospective study reflects our clinical practice over a four-year period and provides information on sedative approaches for EEG studies. Until 2020, we routinely performed EEG recordings using oral melatonin and hydroxyzine. Although most patients successfully completed the exam (89.2%), a significant failure rate was found in children with prominent behavioral problems. This percentage was even higher in patients receiving antipsychotic drugs. To improve sedation effectiveness in this special population, in 2021 we introduced In-Dex sedation for ASD patients with a low level of collaboration undergoing EEG.

In-Dex has been successfully used in the general pediatric population for non-painful procedural sedation, such as EEG recordings (19, 20). More recently, In-Dex sedation proved more efficacious and more tolerable compared to triclophosphate sodium in a group of children with ASD undergoing EEG testing (16).

Since the introduction of In-Dex in our clinical practice, we found a significant reduction in the total number of failures of EEG recordings in patients with severe behavioral disturbances and in those receiving antipsychotic drugs. These findings strongly support the need for both accurate clinical observation and careful parents interview focusing on the presence of severe behavioral disturbance to identify less cooperative patients. In fact, in our opinion, indiscriminate use of In-Dex for all ASD patients would be not appropriate, considering that most ASD children are able to successfully complete the EEG recording receiving milder sedative drugs. Furthermore, In-Dex sedation could be possibly considered as a second choice in patients who initially failed the exam using more traditional sedative protocols. The effectiveness of this approach in our 5 patients who repeated the EEG because of previous failure further supports the usefulness of In-Dex sedation as a rescue sedation strategy.

Our success rate of In-Dex sedation (94.2%) was slightly higher than that observed in other studies on children with ASD. In Kaplan’s study the rate of sedation failure in children undergoing EEG recording receiving 2.95 ± 1.2 mcg/kg In-Dex was 17%. Grau Luque et al. (21) reported a success rate of 77.8% in children sedated using 3 mcg/Kg In-Dex alone and of 92.8% in children receiving oral CH followed by 2 mcg/Kg In-Dex as a rescue to perform auditory brain response hearing tests. It is conceivable that our higher rate of success could be partially explained by the higher In-Dex dose used in our protocol (5 mcg/Kg).

In keeping with previous studies, we did not register any major adverse side effects, confirming Dexmedetomidine optimal safety profile. Specifically, no respiratory depression or hemodynamic disturbances requiring medical intervention were reported. It should also be noted that the administration of In-Dex sedation is fast and easy and requires minimal collaboration from patients. The entire procedure can be completed in a dedicated outpatient space.

This study provides significant insights for clinicians by enhancing our current understanding of sedation in patients with ASD. Children with ASD pose challenges for pediatric anesthesiologists due to various behavioral issues, often leading to referrals for anesthesia services even for minor procedures. Moreover, these patients experience a higher burden of co-occurring medical conditions compared to typically developing children (22), leading to more frequent visits to the emergency department, hospitalization, radiologic imaging, or laboratory tests. Therefore, developing evidence-based behavioral management strategies and pharmacological protocols for safe procedural sedation is essential to optimize care for this vulnerable population.

Our study not only confirms the efficacy and high safety profile of Dexmedetomidine in children with ASD, but also emphasizes the importance of a personalized approach that takes into account the individual characteristics of each patient when selecting the most appropriate sedation method. From this perspective, the direct involvement of parents and caregivers is crucial for identifying the specific needs of patients and implementing tailored responses that allow for an individualized approach.

For children with significant adaptation difficulties and severe behavioral disorders, medical procedures are often experienced as traumatic by both the child and the parents. In this vulnerable population, identifying a sedative agent that is not only effective but also well-tolerated is crucial.

Given its characteristics, In-Dex may be considered a potentially effective alternative for sedation in children with ASD across various clinical settings, including both diagnostic and therapeutic procedures. Among the diagnostic tests, frequently required in the assessment of children with ASD are brain magnetic resonance imaging (MRI), computed tomography (CT) scans, or auditory brainstem response (ABR) tests. Few studies to date have investigated the potential use of In-Dex in these specific settings for the ASD population (21, 23, 24). As shown in studies on the general pediatric population (25), In-Dex may also be considered for patients with ASD for painful procedures, due to its analgesic-sparing effect. Moreover, a recent study suggests its effective use as a treatment for anxiolysis in ASD patients in the emergency department.

Further studies involving larger samples of patients with ASD will be necessary to validate these initial observations. Prospective studies comparing In-Dex with other sedative agents will be essential, especially for assessing the tolerability and efficacy of treatments in different clinical settings.

Our study has some limitations. Data were collected over a long period of time, so that it is more difficult to control for potential confounding variables, although keeping the “biennium of the procedure” in the model as a correction factor contributes to limit this source of bias.

Moreover, two relevant variables in our model, i.e. “intellectual level” and “presence of significant behavioral disturbances”, were dichotomized for analysis purposes. This was of assistance in the context of logistic regression, but clearly imposes constraints on the degree of refinement that can be achieved with regard to the explanatory variables and ORs.

Due to the retrospective non-randomized study design, our results could suffer from selection bias and potential errors in recording or recalling data. More substantially, inferential considerations on such a design should be considered explorative. In this setting the logistic model we build may be a useful suggestion playing the role of a training set that future studies could validate prospectively with an adequate sample size. With respect to this, alternative models such as decision trees, random forests, or neural networks could provide additional insights or enhance predictive accuracy.

In conclusion, in this retrospective study, we demonstrated that children with ASD undergoing EEG recording with In-Dex sedative protocol have a higher successful rate compared to use of melatonin and hydroxyzine alone. The usefulness of this approach is more evident for patients with severe behavioral disturbances and a low level of collaboration. Furthermore, In-Dex sedation has been proven to be both safe and easy to manage. The findings of this study, which reflect real-world practice, may be useful for Pediatric Neurology and Neurophysiology centers that deal with ASD patients.

## Data Availability

The raw data supporting the conclusions of this article will be made available by the authors, without undue reservation.

## References

[1] American Psychiatric Association. Diagnostic and Statistical Manual of Mental Disorders. 5th. Arlington, VA: American Psychiatric Association (2013).

[2] Amiet C Gourfinkel-An I Bouzamondo A Tordjman S Baulac M Lechat P. Epilepsy in autism is associated with intellectual disability and gender: evidence from a meta-analysis. Biol Psychiatry. (2008) 64:577–82. doi: 10.1016/j.biopsych.2008.04.030 18565495

[3] Lukmanji S Manji SA Kadhim S Sauro KM Wirrell EC Kwon CS. The co-occurrence of epilepsy and autism: A systematic review. Epilepsy Behav. (2019) 98:238–48. doi: 10.1016/j.yebeh.2019.07.037 31398688

[4] Luz-Escamilla L Morales-González JA. Association between interictal epileptiform discharges and autistic spectrum disorder. Brain Sci. (2019) 9:185. doi: 10.3390/brainsci9080185 31366163 PMC6721430

[5] Hirosawa T An KM Soma D Shiota Y Sano M Kameya M. Epileptiform discharges relate to altered functional brain networks in autism spectrum disorders. Brain Commun. (2021) 3:fcab184. doi: 10.1093/braincomms/fcab184 34541529 PMC8440646

[6] Ghacibeh GA Fields C. Interictal epileptiform activity and autism. Epilepsy Behav. (2015) 47:158–62. doi: 10.1016/j.yebeh.2015.02.025 25847431

[7] Anukirthiga B Mishra D Pandey S Juneja M Sharma N. Prevalence of epilepsy and inter-ictal epileptiform discharges in children with autism and attention-deficit hyperactivity disorder. Indian J Pediatr. (2019) 86:897–902. doi: 10.1007/s12098-019-02977-6 31123917

[8] Hirosawa T Kikuchi M Fukai M Hino S Kitamura T An KM. Association between magnetoencephalographic interictal epileptiform discharge and cognitive function in young children with typical development and with autism spectrum disorders. Front Psychiatry. (2018) 9:568. doi: 10.3389/fpsyt.2018.00568 30510521 PMC6254014

[9] San-juan D Chiappa KH Cole AJ. Propofol and the electroencephalogram. Clin Neurophysiol. (2010) 121:998–1006. doi: 10.1016/j.clinph.2009.12.016 20071229

[10] Keidan I Ben-Menachem E Tzadok M Ben-Zeev B Berkenstadt H. Electroencephalography for children with autistic spectrum disorder: a sedation protocol. Paediatr Anaesth. (2015) 25:200–5. doi: 10.1111/pan.12510 25145661

[11] Bektas O Arıca B Teber S Yılmaz A Zeybek H Kaymak S. Chloral hydrate and/or hydroxyzine for sedation in pediatric EEG recording. Brain Dev. (2014) 36:130–6. doi: 10.1016/j.braindev.2013.03.002 23582501

[12] Zhang G Xin L Yin Q. Intranasal dexmedetomidine vs. oral midazolam for premedication in children: a systematic review and meta-analysis. Front Pediatr. (2023) 11:1264081. doi: 10.3389/fped.2023.1264081 38027288 PMC10661234

[13] Mason KP O’Mahony E Zurakowski D Libenson MH. Effects of dexmedetomidine sedation on the EEG in children. Paediatr Anaesth. (2009) 19:1175–83. doi: 10.1111/j.1460-9592.2009.03160.x 20017865

[14] Sleigh JW Vacas S Flexman AM Talke PO. Electroencephalographic arousal patterns under dexmedetomidine sedation. Anesth Analg. (2018) 127:951–9. doi: 10.1213/ANE.0000000000003590 29933272

[15] Chen H Yang F Ye M Liu H Zhang J Tian Q. Intranasal dexmedetomidine is an effective sedative agent for electroencephalography in children. BMC Anesthesiol. (2020) 20:61. doi: 10.1186/s12871-020-00978-z 32145737 PMC7060610

[16] Kaplan E Shifeldrim A Kraus D Weissbach A Kadmon G Milkh R. Intranasal dexmedetomidine vs oral triclofos sodium for sedation of children with autism undergoing electroencephalograms. Eur J Paediatr Neurol. (2022) 37:19–24. doi: 10.1016/j.ejpn.2022.01.005 35016051

[17] Aldrete JA. The post-anesthesia recovery score revisited. J Clin Anesth. (1995) 7:89–91. doi: 10.1016/0952-8180(94)00001-k 7772368

[18] Maenner MJ Warren Z Williams AR Amoakohene E Bakian AV Bilder DA. Prevalence and characteristics of autism spectrum disorder among children aged 8 years - autism and developmental disabilities monitoring network, 11 sites, United States, 2020. MMWR Surveill Summ. (2023) 72:1–14. doi: 10.15585/mmwr.ss7202a1 PMC10042614 36952288

[19] Baier NM Mendez SS Kimm D Velazquez AE Schroeder AR. Intranasal dexmedetomidine: an effective sedative agent for electroencephalogram and auditory brain response testing. Paediatr Anaesth. (2016) 26:280–5. doi: 10.1111/pan.12851 26814037

[20] Liu H Sun M Zhang J Tian Q Yu Q Liu Y. Determination of the 90% effective dose of intranasal dexmedetomidine for sedation during electroencephalography in children. Acta Anaesthesiol Scand. (2019) 63:847–52. doi: 10.1111/aas.13372 30982953

[21] Luque CG Atkins-Labelle C Pauwels J Costello R Kozak FK Chadha NK. Intranasal Dexmedetomidine increases the successful sedation of children with autism for out-patient auditory brainstem response hearing tests. Int J Pediatr Otorhinolaryngol. (2021) 151:110945. doi: 10.1016/j.ijporl.2021.110945 34736008

[22] Micai M Fatta LM Gila L Caruso A Salvitti T Fulceri F. Prevalence of co-occurring conditions in children and adults with autism spectrum disorder: A systematic review and meta-analysis. Neurosci Biobehav Rev. (2023) 155:105436. doi: 10.1016/j.neubiorev.2023.105436 37913872

[23] Li BL Yuen VM Zhang N Zhang HH Huang JX Yang SY. A comparison of intranasal dexmedetomidine and dexmedetomidine plus buccal midazolam for non-painful procedural sedation in children with autism. J Autism Dev Disord. (2019) 49:3798–806. doi: 10.1007/s10803-019-04095-w 31172338

[24] Kenneally A Cummins M Bailey A Yackey K Jones L Carter C. Intranasal dexmedetomidine use in pediatric patients for anxiolysis in the emergency department. Pediatr Emerg Care. (2023) 39:685–91. doi: 10.1097/PEC.0000000000002901 36728557

[25] Mondardini MC Amigoni. A Cortellazzi P Di Palma. A Navarra C Picardo SG. Intranasal dexmedetomidine in pediatrics: update of current knowledge. Minerva Anestesiol. (2019) 85:1334–45. doi: 10.23736/S0375-9393.19.13820-5 31630510

